# Teenage Pregnancies in Nepal - The Problem Status and Socio-Legal Concerns – Letter to The Editor

**DOI:** 10.31729/jnma.4406

**Published:** 2019-08-31

**Authors:** Ishan Poudel

**Affiliations:** 1 California Institute of Behavioral Neurosciences & Psychology, 4571 Mangels Blvd, Fairfield, CA 94534, U.S.A.

**Dear Editor**,

It was interesting to read the article Teenage Pregnancies in Nepal - The Problem Status and Socio-Legal Concerns.^1^

This article indeed takes consideration of scenario of teenage pregnancies in major health care facilities of Nepal. We appreciate the author's effort to draw attention of readers that may also include the Health Policy makers of Nepal regarding legal age of marriage and teenage or adolescent pregnancies. It is obvious from the article that authors have made a considerable effort in identifying the number of teenage pregnancies occurring within a marriage and outside of marriage.^1^ Authors have also stated that they analyzed the data to include the number of adolescent mothers who are less than 20 years of age at the time of delivery.^1^

Nepal stands to have open borders with the neighboring nation of Union of India.^2^ Healthcare facilities are widely utilized by citizens of both the nations on each other's land. The authors do not clearly state the nationality of the participants of the study. The residence of the participants also remains a major factor as people of both Indian and Nepalese nationality have been living within each other's community. People who officially write their nationality as Nepalese have been living most of their lives in India and vice versa.

So, if the above scenario is the case, it is utterly unfair to project the data on behalf of Nepal and people living in Nepal who have a distinct cultural and socio-economic background.

## Conflict of Interest:

**None.**
